# Simple Dressing by Continued Moisture

**Published:** 1871-01

**Authors:** 


### SIMPLE DRESSING BY CONTINUED MOISTURE.

Dr. Leon le Fort, in an article bearing this title, translated from the *Boston Medical and Surgical Journal*, says:

If we seek the indications which surgeons have attempted to fulfil by their various dressings, we find them as follows:

To exclude the air.

To change the condition of the wound, when expedient, by medicated dressings.

To maintain a certain degree of moisture.

To prevent decomposition of the pus taken up by the dressing.

To keep the wound clean.

To prevent the adhesion of the dressings.

To destroy germs which might be the source of infection.

A slight modification of the methods usually employed has enabled me, as I believe, to fulfil these various indications, as already stated. I have absolutely rejected all fatty agents whatever, and I extend the same proscription to diachylon, so far as fresh wounds are concerned; and in no case, at least in hospitals, do I use lint, because by the power of absorption it becomes the ready receptacle of infectious germs. I cover the wound with one or more compresses, saturated with a mixture containing one part of alcohol or camphorated alcohol, and nine parts of water; if the wound needs stimulating I add, according to the necessities of the case, a tenth part of a solution of sulphate of zinc. Over all I place a piece of oiled silk, kept in position by a few turns of bandage; and I take care that this covering shall be tight and entire. The evaporation of the fluid with which the compress is filled cannot progress, and the insensibe perspiration, which occurs normally on the surface of the skin, being retained, the dressing becomes converted into a sort of continued bath.

Without the inconveniences of a maceration which distends the tissues and seems to lessen their vitality, without the annoyances caused by the necessity to use an apparatus applied with difficulty, I get the advantage of the bath of Mayor, Langenbeck, and Valette, and those indeed of continued irrigation. The sedative effect of the water, modified according to necessity by the use of medicated solutions, controls the inflammation and keeps it within the bounds necessary to the process of cicatrization. The pus, excluded from the air, undergoes no change; it remains indeed about the wound, but the air-tight dressing showed us long ago the harmlessness of unaltered pus. The compresses cannot dry and adhere and are easily removed, and there is no fear of bruising the granulations. As regards cleanliness, it is seen at once to be absolutely attained. Finally, with respect to infection and the transportation of germs, the wound, being wet at the outset with alcoholized water, covered with compresses filled with the same fluid, and enclosed hermetically in an impermeable tissue, is fully protected from all contamination. This innovation upon a dressing in such general use, consisting essentially in the employment of a piece of oiled silk larger than usual, presents such an appearance of insignificance that I should have hardly dared to introduce it if it were not recommended by results which have convinced me of its efficacy. —*Medical Archives.*

